# Concussion research at the National Institutes of Health: an update from the National Institute of Neurological Disorders and Stroke

**DOI:** 10.2217/cnc.15.10

**Published:** 2015-10-26

**Authors:** Meghan Mott, Walter Koroshetz

**Affiliations:** 1 National Institutes of Health, National Institute of Neurological Disorders & Stroke, Bldg 31, Room 8A52, 31 Center Dr. MSC 2540, Bethesda, MD 20892, USA

**Keywords:** mild traumatic brain injury, post concussive symptoms, sports head injury

## Concussion: the scope of the issue

Concussion is an exceedingly common type of traumatic brain injury (TBI), yet little is known about what happens to the brain at the time of concussion. A concussion can be defined as a sudden onset change in neurologic function that occurs immediately after the brain encounters a mechanical force. When the head sustains an injury, direct or rotational biomechanical forces induce physiologic dysfunction; the most characteristic is immediate loss or alteration of consciousness. Each year, between 1.6 and 3.8 million sports-related concussions occur in the USA, particularly in youth athletes [1]. In the US military, it is estimated that roughly 20% of the deployed forces suffered a head injury in the wars in Iraq and Afghanistan, 83% of whom endured a mild, uncomplicated TBI or concussion [2]. While striking, these figures are likely vast underestimates of the actual number of afflicted individuals, since many who suffer concussion do not seek medical attention.

The issues surrounding concussion can be divided into three major areas:

- What is the mechanism of concussion? Common symptoms associated with concussion include rapidly resolving loss of consciousness, headache, confusion, memory impairment, attention and executive function, sense of dizziness, imbalance, sleep disturbances and behavioral changes.
- What is the mechanism of recovery from concussion? Most commonly, concussive symptoms improve over a period of hours, days or weeks. In some patients, however, symptoms persist, known as postconcussive syndrome.
- What are the longer-term or even permanent effects of repeated concussion? Repeated concussion presents other problems: more prolonged recovery period with subsequent concussions, and the risk of a delayed neurodegenerative disorder call chronic traumatic encephalopathy (CTE). In addition, a rare condition has been described in which fatal or near-fatal, rapid onset, brain swelling occurs with concussion. It has been questioned whether the latter occurs due to a second concussion occurring before the brain's autoregulation of blood flow has recovered from a preceding concussion, so-called ‘second hit’ phenomenon.

Most of the key questions in these three areas of concussion research remain to be solved. We know little of the underlying mechanisms and pathophysiology of concussion and postconcussive syndrome, as well as the vulnerability and mechanisms of CTE.

## Chronic traumatic encephalopathy

The most devastating consequence of multiple, repetitive concussion is a progressive degenerative disease in the brain, known as CTE. In its end stage, it causes dementia, severe brain atrophy with confluent, widespread, intraneuronal tau aggregates. Only recently have there been studies of cases with intermediate levels of tau aggregates in the brain; the majority of these have been professional American football players. Indeed cases with limited, multifocal tauopathy in neurons and astrocytes have also been described and linked to history of repeated concussion. The clinical syndrome associated with the latter cases is not entirely clear as the data are biased by the situation that led to the person's death and subsequent CTE diagnosis on autopsy examination. Inferring causal link between the pathology and the symptoms is biased by the fact that diagnosis is based on examination of brain tissue from young or middle-aged individuals who have died of other causes. Death due to suicide or drug overdose have been prominent in the CTE brain tissue repositories, raising the question of whether depression and suicidality is a core feature of early-stage CTE or coincidental. Understanding the characteristics of the clinical syndrome, its prevalence, relationship to specific concussive exposures, individual differences in vulnerability and best treatment will depend on developing a means of detecting the pathology in living individuals. There is much enthusiasm that the tau radioligands currently under study will enable the ability to diagnose at least the late and intermediate stages of CTE.

## NIH investment in TBI research

The National Institute of Neurological Disorders and Stroke (NINDS) leads TBI research at NIH, which encompasses the full range of TBI severity, from mild (concussion) through severe TBI (car crash or serious fall). NINDS supports and conducts research on the mechanisms of immediate and delayed damage to the brain and of recovery, devising better diagnostic tools and developing more effective treatments. Research ranges from laboratory studies in animal models through clinical trials. In addition, fundamental NIH research on brain structure and function, including major initiatives such as the Human Connectome Project and The BRAIN Initiative^SM^, provides the foundation for understanding the effects of TBI on people.

The NIH funded $87 million of research on TBI in FY 2014. The National Institute on Child Health and Human Development (NICHD) supports research on pediatric TBI and coordinates NIH rehabilitation research through the National Center for Medical Rehabilitation Research. NIH is currently funding two large TBI clinical research programs in the USA to collect data to determine what treatments work best for which patients with TBI. One of these clinical trials, Approaches and Decisions in Acute Pediatric TBI [6], focuses on treatment effectiveness in 1000 children with severe TBI, while the other. The other, Transforming Research and Clinical Knowledge in TBI [7], focuses on effective treatments in 3000 adults with TBI across the spectrum of injury severity. These trials are coordinated with studies in Europe and Canada through the International Initiative for TBI Research [8], a cooperative effort to advance clinical TBI research, treatment and care globally. NIH and the Department of Defense (DOD) together lead the Federal Interagency Traumatic Brain Injury Research [9] Informatics System, a $10 million project funded by the DOD to build a central repository and resource for sharing data to promote collaboration, accelerate research and advance knowledge on the characterization, prevention, diagnosis and treatment of TBI. Together with the Uniformed Services University of the Health Sciences (USUHS), and the Walter Reed National Military Medical Center, the NIH Intramural Research Program founded the Center for Neuroscience and Regenerative Medicine [10], a collaborative federal intramural research program that brings together clinicians and scientists across disciplines to catalyze innovative approaches to TBI research.

## NIH/NFL Sports & Health Research Program

In 2012, the National Football League (NFL) donated $30 million to the Foundation for the NIH to launch the Sports and Health Research Program (SHRP), a public–private partnership which augments ongoing research on TBI and other sports injuries [11]. Through a series of workshops and Funding Opportunity Announcements, SHRP has made it possible for NIH researchers to begin to unravel the complex neuropathology of TBI, as well as develop new methods of detection, intervention and rehabilitation in concussion research. In December 2012, 60 scientists came together at NIH to identify the CTE neuropathology and prioritize research challenges and strategies to fill critical gaps in knowledge [12].

In March 2013, NIH released a Request for Applications and selected two 4-year projects, each multicenter study was funded at $6 million, aimed at studying the neuropathology associated mild TBI and CTE, and the delayed effects of TBI, in order to identify neuroimaging signatures of the neuropathology as a foundation for the development of *in vivo* diagnostic tools. The program has funded two large cooperative programs to understand the delayed neurodegeneration that may occur in the brains of people exposed to single and repetitive concussions and to develop diagnostics that detect these effects in living people.

The SHRP program also funded six pilot projects to improve diagnosis of concussion on the sidelines and identify markers that can better track concussion injury and recovery. More about each of the six pilot projects funded by SHRP can be found online [13]. These studies focus on improving the diagnosis of concussion and identifying potential biomarkers that can be used to track a person's recovery. The SHRP program is helping researchers get closer to answering some of the important questions about concussion for athletes, and to extend the impact of that research beyond the playing field to benefit others, including the brave members of our military.

In February 2015, NIH sponsored the first consensus conference to define criteria for the pathological diagnosis of CTE by bringing together nine neuropathology experts to review the literature and relevant pathologic cases, and ultimately to develop recommendations [14]. In CTE, the tau lesion considered pathognomonic was an abnormal perivascular accumulation of tau in neurons, astrocytes and cell processes in an irregular pattern at the depths of the cortical sulci. Many other abnormalities were seen, especially in the more severely affected brains, but the group consensus was that abnormal tau immunoreactivity in neurons and glia, in an irregular, focal, perivascular distribution and at the depths of cortical sulci, was required for the diagnosis of CTE. Recommendations were also made for conducting a neuropathologic examination for CTE. They also discussed how extensive the sampling must be to rule out CTE, tissue blocks, including the sulcal depth from superior and middle frontal gyrus, superior and middle temporal gyrus and inferior parietal gyrus, were considered to be most informative for detecting the earliest or most mild lesions of CTE.

The group described several required and supportive criteria in the process of fully characterizing the neuropathology of CTE, and this workshop represents the first of a series of consensus conferences of investigators funded by the NIH research initiative. Significantly, the investigators noted that thus far, the CTE pathology has only been found in individuals exposed to brain trauma, typically multiple episodes. How common this pathology occurs at autopsy and the nature and degree of trauma necessary to cause this neurodegeneration remain to be determined.

The combined results of these two neuropathologic studies promise to answer critical questions about the chronic effects of single versus repetitive injuries on the brain, how repetitive TBI might lead to CTE, how commonly these changes occur in an adult population, and how CTE relates to neurodegenerative disorders like Alzheimer's disease. Building off of these pathologic studies, NIH, with funding support from the SHRP, has solicited applications for research in living patients with neuro/psychiatric symptoms with high-risk exposures for CTE in order to characterize the clinical syndrome and its progression over time.

## Future directions in concussion research

Although NIH-funded researchers have made significant strides in concussion and TBI research through programs like SHRP, fundamental questions remain. What dose of TBI (e.g., number, intensity, temporal pattern, regional factors) is associated with subsequent foci of tau deposits in the brain? In CTE, how does the tau deposition evolve to affect widespread regions? Given similar exposures, can we predict an individual's risk for CTE using genetics, environmental influences and/or lifestyle? Moving forward, large longitudinal cohort studies will be necessary to begin to uncover the answers.

Independent of the CTE issue, there is a knowledge gap in understanding the mechanisms and consequences of concussion in our youth. Parents need to make data-driven decisions about their child's participation in contact sports. We know little about the consequences of concussion on scholastic performance, behavioral and social maturation or the risk associated with return to play and potentially suffering further concussion. The incipient NIH Adolescent Brain Cognitive Development (ABCD) study, for example, offers a unique opportunity to follow 10,000 youth starting at age 9 years for a decade to study their brain development [15].

NINDS is committed to research on the impact of concussion on the brain at all ages in all people. Our mission in TBI research is to develop strategies and interventions to limit the primary and secondary brain damage that occurs as a result of head trauma, and to devise therapies to treat brain injury and improve long-term recovery of function. Achieving this mission not only will require clinical studies of affected individuals but also will require bright scientists to study the basic mechanisms of concussion and brain recovery after concussion.
